# Atypical Presentation of a Cerebellar Abscess Caused by Streptococcus intermedius: Importance of Early Recognition

**DOI:** 10.7759/cureus.101892

**Published:** 2026-01-20

**Authors:** Iker F Garcia Contreras, César Vega-López, Daniel A Seniscal Arredondo, Yuscely Flores Jurado, Sandra Rivera

**Affiliations:** 1 Internal Medicine, Hospital Angeles Pedregal, Mexico City, MEX; 2 Infectious Diseases, Hospital Angeles Pedregal, Mexico City, MEX

**Keywords:** brain infection, cerebellar abscess, infectious disease, magnetic resonance, streptococcus anginosus

## Abstract

A cerebellar abscess due to *Streptococcus intermedius* is an uncommon clinical entity that may present with features resembling a neoplastic lesion, making early recognition challenging. *S. intermedius,* a member of the *Streptococcus anginosus* group, has a marked ability to induce tissue destruction and trigger a pronounced inflammatory response, ultimately leading to the formation of purulent collections with peripheral enhancement on neuroimaging. Although gram-positive cocci are recognized causes of brain abscesses, involvement of the cerebellum by this organism is rare. Awareness of this atypical clinical presentation is critical, since its pseudotumoral behavior may obscure the underlying infectious etiology and delay appropriate therapy. Integrating clinical manifestations, radiological characteristics, and microbiological confirmation is therefore essential for timely diagnosis and favorable clinical outcomes.

## Introduction

A cerebellar abscess secondary to *Streptococcus intermedius* may follow a pseudoneoplastic pattern, both clinically and radiologically, thereby complicating the initial diagnostic approach. *S. intermedius*, a member of the *Streptococcus anginosus* group, is characterized by its marked tissue tropism and its ability to induce suppuration. Among its virulence determinants, the expression of antigen I/II-type surface proteins stands out, facilitating adhesion to the extracellular matrix components such as fibronectin and laminin, while promoting a local inflammatory response through IL-8 induction and neutrophil recruitment [1,2]. This inflammatory cascade contributes to the development of lesions with peripheral enhancement and vasogenic edema, features that can be indistinguishable from primary brain neoplasms in early stages [1,2].

Several risk factors for brain abscess formation have been identified, including odontogenic, sinus, and otogenic sources in 29.8% of cases, and intravenous drug use in 14.9%. However, no underlying source is found in 44.7% of patients. It is noteworthy that only 4% of cases involve the cerebellum [3,4]. Indeed, only a minority of brain abscesses are reported to arise in this location [3]. Within the* Streptococcus anginosus* group, *S. intermedius *is particularly notable for its invasiveness and marked pyogenic capacity, exhibiting the highest rate of abscess formation and a greater predilection for the central nervous system than other group members [1,2,5].

In this context, the absence of typical symptoms or combination of headache, fever, nausea or vomiting, gait instability, and signs of posterior fossa involvement, elevated inflammatory markers and imaging findings suggestive of an infectious process made hard the initial diagnosis, so recognizing the pseudoneoplastic behavior of cerebellar abscesses, in this case, caused by *S. intermedius* is essential, as delays in suspecting an infectious etiology may lead to significant postponement of targeted treatment and ultimately worse neurological outcomes [1-5].

## Case presentation

A 63-year-old woman presented with a two-week history of nausea and vomiting, followed by gait instability, headache, and right-sided limb incoordination. On admission, her vital signs were as follows: BP 134/89 mmHg, HR 76 bpm, RR 18 rpm, SpO₂ 92%, and T 36°C. She exhibited dysmetria, mild dysarthria, and a scanning speech pattern. Laboratory evaluation showed a white blood cell count of 9.6 ×10³/µL (reference range: 3.8-11.20 ×10³/µL) with relative neutrophilia (86.7%; reference range: 40-70%; absolute neutrophil count 8.32 ×10³/µL; reference range: 1.50-7.80 ×10³/µL) and a mildly elevated high-sensitivity C-reactive protein of 6.5 mg/L (reference range: <5.0 mg/dL). Hemoglobin (16.8 g/dL; reference range: 14-18 g/dL)) and platelet count (244 ×10³/µL; reference range: 130-400 ×10³/µL) were within normal ranges. Renal function revealed a serum creatinine of 1.24 mg/dL (reference range: 0.70-1.30 mg/dL) with an estimated glomerular filtration rate of 45.26 mL/min/1.73 m² (reference range: 70-110 mL/min/1.73 m²). Given the absence of systemic signs of infection, blood cultures were not obtained at the time of presentation.

**Figure 1:**
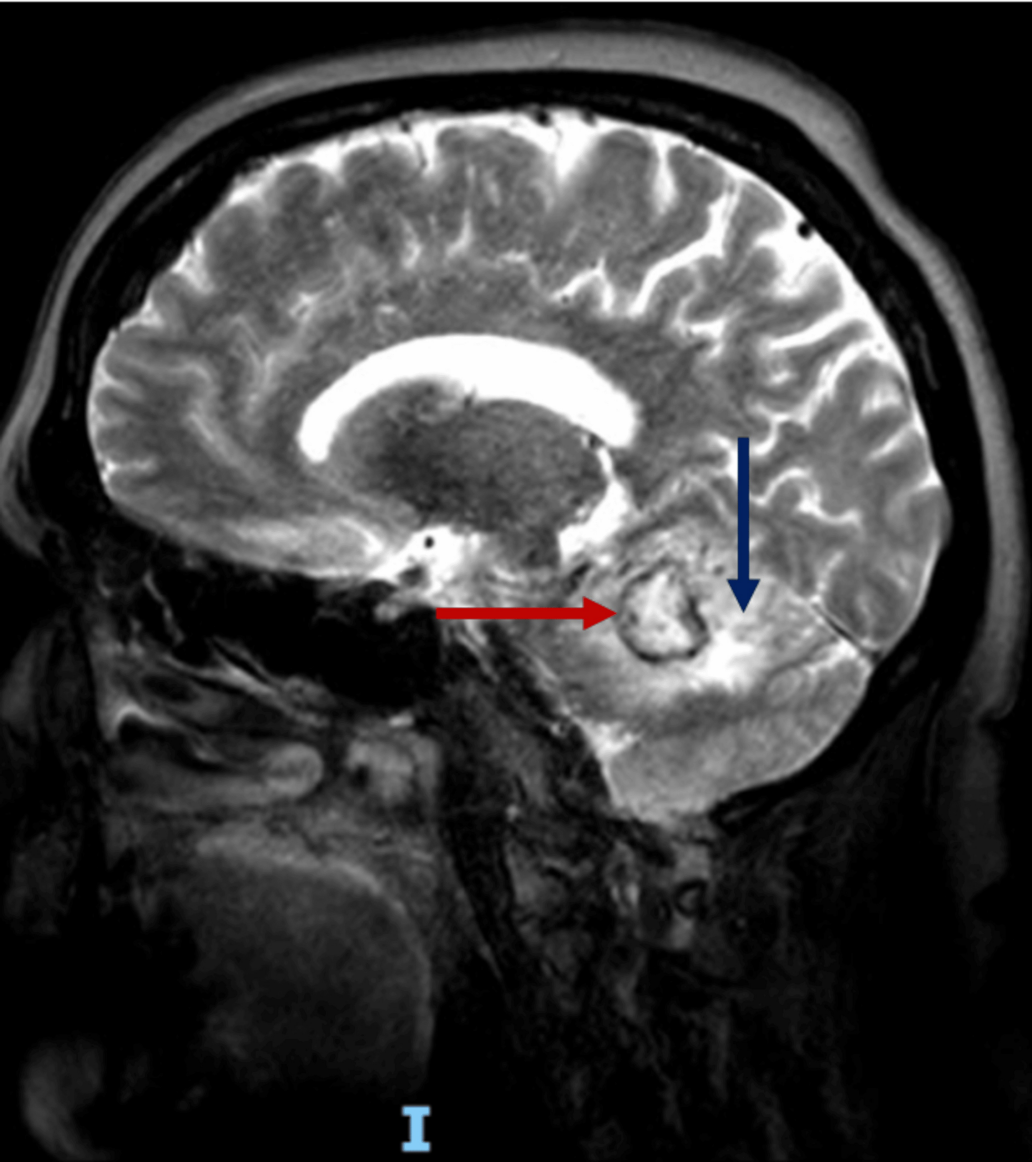
T2-weighted brain magnetic resonance image Ring-enhancing lesion (red arrow) in the right cerebellar hemisphere with edema extending toward the right fifth cranial nerve and the ipsilateral recess of the fourth ventricle (blue arrow)

Brain magnetic resonance imaging (Figure 1) revealed a right cerebellar hemispheric lesion with heterogeneous diffusion restriction. On T2-weighted images, the lesion demonstrated a peripheral ring with suspected hemosiderin deposition and surrounding vasogenic edema. Magnetic resonance spectroscopy showed a prominent lipid peak, without lactate elevation. Choline, creatine, and N-acetylaspartate peaks were preserved, and no alanine, succinate, or cytosolic amino acid peaks were identified. Based on these findings, the radiological impression favored a space-occupying cerebellar lesion to rule out cavernous angioma.

Due to rapid enlargement of the lesion within less than 24 hours, despite the absence of other symptoms, a right lateral suboccipital craniectomy was performed, revealing a purulent collection consistent with a cerebellar abscess. No fever episodes were documented before or after hospital admission. 

Empirical antimicrobial therapy was initiated with ceftriaxone 2 g every 12 hours, vancomycin dosed according to the patient’s actual body weight, and metronidazole 500 mg every eight hours targeting gram-positive cocci, enteric gram-negative bacilli, and strict anaerobes, given suspected hematogenous dissemination. Culture of the lesion (Table 1) identified Streptococcus intermedius belonging to the *S. anginosus* group, prompting adjustment to monotherapy with ceftriaxone. The patient showed favorable clinical evolution with resolution of the infectious process, allowing discharge following completion of four weeks of antibiotic treatment, rehabilitation and neuroimaging postprocedure, also no motor or visual deficits were present.

**Table 1:** Antimicrobial susceptibility profile of Streptococcus intermedius The organism demonstrated susceptibility to all beta-lactams, fluoroquinolones, and protein synthesis inhibitors, with resistance observed only to clindamycin.

Antibiotic	MIC (µg/mL)	Interpretation
Ampicillin	≤ 0.25	Susceptible
Cefotaxime	≤ 0.5	Susceptible
Ceftriaxone	1	Susceptible
Clindamycin	≥ 1	Resistant
Chloramphenicol	2	Susceptible
Levofloxacin	≤ 0.25	Susceptible
Linezolid	< 2	Susceptible
Moxifloxacin	≤ 0.25	Susceptible
Tetracycline	≤ 0.06	Susceptible
Tigecycline	0.06	Susceptible
Penicillin G	0.12	Susceptible

## Discussion

This case illustrates a cerebellar abscess caused by *S. intermedius*, presenting with pseudotumoral behavior, exerting mass effect and producing an atypical clinical picture of the patient, underscoring the need to consider this etiology in the differential diagnosis of rapidly enlarging cerebellar lesions [1,3-5]. Although gram-positive cocci are well-recognized agents of brain abscesses, cerebellar involvement by *S. intermedius* is rare and may go unrecognized if the lesion is initially interpreted as neoplastic [1-4].

The intrinsic virulence of *S. intermedius* promotes tissue destruction and the formation of purulent cavities with peripheral enhancement and vasogenic edema on magnetic resonance imaging. These features may mimic primary tumors or metastatic lesions [1,3,4]. This radiological similarity explains why, in early stages, the diagnosis may be directed toward neoplasia, particularly when the clinical presentation is subacute and no obvious infectious source is identified.

Brain abscesses pose a diagnostic challenge due to nonspecific manifestations, and their recognition depends on integrating clinical, neuroimaging, and microbiological data. Contemporary series report that up to one-third of patients require diagnostic surgical resection when imaging fails to provide clarity [6,7]. Obtaining material for culture remains a cornerstone of management, allowing identification of the causative pathogen, antimicrobial susceptibility testing, and avoidance of inappropriate therapy [1-4,6].

Multiple studies emphasize that optimal management of central nervous system abscesses involves surgical drainage combined with bactericidal antibiotics with sufficient penetration into neural tissue [6-8]. In infections due to *S. intermedius*, early administration of parenchyma-penetrating beta-lactams, such as third-generation cephalosporins, has demonstrated high rates of microbiological cure and neurological recovery [3,6]. Antimicrobial susceptibility patterns of *S. intermedius* have important therapeutic implications. Although isolates are typically susceptible to β-lactam antibiotics such as ceftriaxone and agents like vancomycin, increasing rates of resistance to macrolides and clindamycin have been reported, underscoring the importance of susceptibility-guided therapy [9]. This consideration is particularly relevant in the management of brain abscesses, where inadequate antimicrobial coverage may adversely affect outcomes.

Recent guidelines further support prolonged antimicrobial courses (typically 4-6 weeks or longer) and structured follow-up with serial imaging because of the risk of recurrence or delayed clinical progression [6-8]. In lesions with rapid expansion or significant mass effect on brainstem structures, early neurosurgical intervention is particularly relevant to prevent irreversible neurological deterioration [7].

## Conclusions

The pseudoneoplastic behavior of cerebellar abscesses caused by *S. intermedius* mandates inclusion of this entity in the differential diagnosis of ring-enhancing cerebellar lesions with surrounding edema, even in the absence of an identifiable infectious focus or even if the patient doesn't develop a febrile syndrome compatible with typical neuroinfection syndrome.

Systematic integration of clinical, radiological, and microbiological information allows timely management and mitigates complications. This case reinforces the importance of early clinical suspicion and the need for responsive diagnostic protocols leading to drainage and targeted antimicrobial therapy, with favorable impact on survival and neurological outcomes.
